# Clarifying “chronic primary musculoskeletal pain”? The waters remain murky

**DOI:** 10.1097/PR9.0000000000001021

**Published:** 2022-08-09

**Authors:** Milton L. Cohen

**Affiliations:** School of Clinical Medicine, St Vincent's Healthcare Clinical Campus, Faculty of Medicine and Health, UNSW Sydney, Australia

## Abstract

**Commentary on**: Fitzcharles M-A, Cohen SP, Clauw DJ, Littlejohn G, Usui C, Häuser W. Chronic primary musculoskeletal pain: a new concept of non-structural regional pain. PAIN Reports 2022;7:e1024.

**See also**: Treede R-D. Chronic musculoskeletal pain: traps and pitfalls in classification and management of a major global disease burden. PAIN Reports 2022;7:e1023.

The publication in this issue of PAIN Reports of the article by Fitzcharles et al.^4^ entitled “Chronic primary musculoskeletal pain: a new concept of non-structural regional pain” is a bold invitation to debate. The article exemplifies the collision, conflation, and competition that can accompany different ways of seeing the world. The question arises, to what extent are these different perspectives equally valid?

First, however, I must draw readers' attention to the mutation of the title of the article, from “Chronic primary musculoskeletal pain: a new concept of regional pain” to “Chronic primary musculoskeletal pain: a new concept of *non-structural* regional pain” (emphasis added here). Although there would be little difficulty in understanding what is meant by “regional” pain—that which is experienced in a region of the body rather than in a discrete or a widespread distribution—the question must be asked, “What is ‘nonstructural’ pain,” regional or otherwise? Not only do the authors not define it but also this is but one example of their looseness with terminology.

In this article, the authors make many assertions of which I would highlight 3 here:

(1) that “… these [primary] pain conditions … can be categorized as (*sic*) a mechanistic pain descriptor termed nociplastic pain”;
(2) that “… chronic primary musculoskeletal (MSK) pain is best understood as ‘regional fibromyalgia’…”; and
(3) that “Regional MSK complaints are commonly recognized as myofascial pain syndromes (MPS)…”.

In dissecting these assertions, I identify that the authors have conflated 3 themes:

(1) a taxonomic category, “primary musculoskeletal pain”;
(2) a clinical descriptor, “nociplastic pain,” that incorporates an hypothesis of (somatic) mechanism (viz, altered central nociceptive function); and
(3) a clinical syndrome of “regional fibromyalgia,” a back derivation from what the authors style as the “nonregional prototype fibromyalgia.”

A taxonomy, in this case ICD-11, is a classification system that “provides a common language that allows health professionals to share standardized information across the world.”^5^ A major innovation in ICD-11 is the recognition of chronic pain as a taxonomic entity in its own right. To quote from the signal publication that introduced the IASP classification of chronic pain for ICD-11^11^:

“Chronic pain is the ‘parent code’ for 7 other codes that comprise the most common clinically relevant groups of chronic pain conditions: (1) chronic primary pain, (2) chronic cancer-related pain, (3) chronic postsurgical or posttraumatic pain, (4) chronic neuropathic pain, (5) chronic secondary headache or orofacial pain, (6) chronic secondary visceral pain, and (7) chronic secondary musculoskeletal pain.”

In ICD-11, chronic primary pain is further subdivided into chronic widespread pain, complex regional pain syndromes, chronic primary headache and orofacial pain, chronic primary visceral pain, and chronic primary musculoskeletal pain. The article by Fitzcharles et al. concerns the last of these.

These authors assert:

"Chronic primary MSK pain now introduces the concept that not all regional pain conditions are solely due to tissue abnormalities but that some aspects can be mechanistically explained as sensitization of the nervous system."

However, the companion signal paper that introduces “chronic primary pain”^9^ does no such thing. First, the essence of chronic primary pain, as a taxonomic entity, is that the pain “cannot be better accounted for by another chronic pain condition.” Second, that paper makes no claim as to possible mechanisms. Indeed, it is stated, “However, at this stage, the relationship of nociplastic pain mechanisms and chronic primary or secondary pain syndromes cannot be determined.”

In neither of these 2 signal papers^9,11^ is the term “regional pain conditions” used; the only use of “regional” in each is with respect to “complex regional pain syndrome” which is not the subject of the present discussion. Fitzcharles et al. can thus be seen to have taken a series of major liberties in extrapolating from “chronic primary musculoskeletal pain” via “[chronic primary] regional musculoskeletal pain” to “[chronic primary] regional pain” (if not also to “[chronic primary] nonstructural regional pain”). In doing so, they seek to resurrect a proposal of “regional fibromyalgia”^8^ as a subset of “the nonregional prototype fibromyalgia.” But of what is “fibromyalgia” the “prototype”?

The authors go on to appropriate, uncritically, the proposals of Kosek et al.^7^ for *identifying* nociplastic pain in the musculoskeletal system to the “*diagnosis* of chronic primary MSK pain” (emphasis added here). They do state that those proposals “still require[d] validation” but fail to acknowledge objections to them.^1^

It must be emphasised that “nociplastic” is a place-holder term that reflects that, clinically, the pain is neither nociceptive nor neuropathic in mechanism *and* that there are features suggesting altered central nociceptive function.^6^ Such altered function may turn out to be “caused” by central sensitisation of nociception (from a “bottom-up” point of view) but may equally be “caused” by hypervigilance (from a “top-down” point of view). It follows that the authors' other implication that “nociplastic” is synonymous with “central sensitization” is as unsustainable as the other conflations mentioned.

A surprising aspect of this paper is the authors' resurrection of “MPS” which, it seems, they seek to rebadge also as “chronic primary musculoskeletal pain.” They acknowledge that, “This syndrome [MPS] is further fraught with controversy as there is currently no universal consensus on the aetiology, pathology, diagnostic criteria, or ideal treatment.” However, not only has MPS been comprehensively refuted as a construct but also the phenomena that it purported to explain are better understood as reflecting altered central nociceptive function.^10^ One cannot argue, however, with their concern about the “persistent misdiagnosis of MPS” and the adverse therapeutic consequences that ensue.

The authors do not answer their own question, “Is chronic primary musculoskeletal pain different from MPS?” But they do undertake a curious manoeuvre, to imply that MPS—now for them to be absorbed by “chronic primary MSK pain”—should be rebadged further as “regional fibromyalgia.” However, “fibromyalgia” is itself also a misnomer, as there is no evidence that the “-algia” originates in “fibromy-” tissues.

This confusion is further exemplified in the Table, which I understand is similar to one published elsewhere.^3^ This table is intended to distinguish between “secondary musculoskeletal pain—predominantly nociceptive” and “primary musculoskeletal pain—predominantly nociplastic.” The conflation here between taxonomy and mechanism is blatant. Furthermore, invoking features to make this distinction that have nothing to do with pain or nociception, such as “diagnostic tests,” “quality of life changes,” and “concomitant conditions,” is a major epistemological error. By ignoring its own caveat of “Categories (*sic*) subject to significant heterogeneity and variability,” this formulation implies certainty that is not justified.

It does seem that the authors are attempting to assert the primacy of the clinical construct of “(regional) fibromyalgia” over the ICD-11 concept of “primary musculoskeletal pain.” They write, “Despite distinct phenotypic differences, the classification of primary MSK pain, with a predominant nociplastic mechanism, may be easiest understood as ‘regional fibromyalgia’…”. Yet later in the same paragraph they write, “… we must unite in support for the concept of chronic primary MSK pain….” Is this trying to ride not 2 but 3 horses simultaneously?

This is a complex area where words matter, concepts collide, and reputations are threatened. Are these issues just semantic or pedantic, or do they reflect fundamental factual differences and does this matter clinically? This debate amplifies a major deficiency in our clinical nomenclature which, despite the advances in ICD-11, continues to incorporate the use of “pain” in pseudodiagnostic labels for conditions characterised by the symptom of pain. For example, to “diagnose” a person presenting with chronic low back pain as having the condition “low back pain” is, frankly, nonsensical. Following ICD-11, previously so-called “nonspecific” low back pain would now be classified as a subset of “chronic primary musculoskeletal pain,” consistent broadly with the aetiological concept of “not explainable by another diagnosis.” It would follow that “chronic primary regional musculoskeletal pain” could also be an acceptable label for taxonomic if not also clinical purposes. But to assert that this is synonymous with “chronic nociplastic regional musculoskeletal pain” which identifies a hypothesis of pathogenesis rather than aetiology, or with “regional fibromyalgia” which denotes a syndrome—that is, pain plus other features—is an epistemological bridge-too-far. The pain literature is replete with misnomers and logical fallacies^2^ and for some epistemic discipline to be applied here is in the interests of all parties, especially our patients.

As a catalyst for debate, this article by Fitzcharles et al. is a sufficient starter. I suspect, however, that the reader will be confused by the authors' repeated conflation of the 3 themes identified above and the lack of coherence of the argument presented. Furthermore, the unsupported introjection of “nonstructural regional pain” further muddies rather than clarifies the murky waters in which diagnostic terminology for pain conditions still floats. The ICD-11/IASP concept of “chronic primary musculoskeletal pain” is the new integrative thinking in this area, although much development is required to bring administrative and clinical usage more closely together. “Regional fibromyalgia” and “myofascial pain syndrome”, both misnomers, have been supplanted by it.

## Disclosures

The author has no conflict of interest to declare.
